# Supplementary material for: Efficient sampling and noisy decisions
Source: eLife. 2020 Sep 15;9:e54962. doi: 10.7554/eLife.54962 (PMC7492090; doi:10.7554/eLife.54962)
Supplement: Transparent reporting form [file elife-54962-transrepform.pdf]

eLIFE
1st Floor
24 Hills Road
Cambridge CB2 1JP, UK
P 01223 855340
W elifesciences.org
T @elife

eLife's transparent reporting form

We encourage authors to provide detailed information *within their submission* to facilitate the interpretation and replication of experiments. Authors can upload supporting documentation to indicate the use of appropriate reporting guidelines for health-related research (see EQUATOR Network), life science research (see the BioSharing Information Resource), or the ARRIVE guidelines for reporting work involving animal research. Where applicable, authors should refer to any relevant reporting standards documents in this form.

If you have any questions, please consult our Journal Policies and/or contact us: editorial@elifesciences.org.

Sample-size estimation

- You should state whether an appropriate sample size was computed when the study was being designed
- You should state the statistical method of sample size computation and any required assumptions
- If no explicit power analysis was used, you should describe how you decided what sample (replicate) size (number) to use

Please outline where this information can be found within the submission (e.g., sections or figure legends), or explain why this information doesn't apply to your submission:

Full details about sample-size estimation and power analyses can be found in the methods subsection 'Power analyses and model recovery' (p. 21, main text).

Replicates

- You should report how often each experiment was performed
- You should include a definition of biological versus technical replication
- The data obtained should be provided and sufficient information should be provided to indicate the number of independent biological and/or technical replicates
- If you encountered any outliers, you should describe how these were handled
- Criteria for exclusion/inclusion of data should be clearly stated
- High-throughput sequence data should be uploaded before submission, with a private link for reviewers provided (these are available from both GEO and ArrayExpress)

Please outline where this information can be found within the submission (e.g., sections or figure legends), or explain why this information doesn't apply to your submission:

eLIFE
1st Floor
24 Hills Road
Cambridge CB2 1JP, UK
P 01223 855340
W elifesciences.org
T @elife

No participants were excluded from the analyses of the data (information provided in *Methods – Participants* section, p. 20 main text)

Each experiment was conducted once. However, the main conclusion of our work was replicated across 3 experiments, each with a different set of participants.

Regarding data exclusion, trials in which the participant blinked or move their gaze too far away from the fixation cross during the stimulus presentation were excluded. Using this criterion resulted in the exclusion of only 212 out of 45,600 trials, i.e., < 0.5% of the trials. Full details are provided in *Methods – Apparatus* section (p. 22 main text)

Statistical reporting

- Statistical analysis methods should be described and justified
- Raw data should be presented in figures whenever informative to do so (typically when N per group is less than 10)
- For each experiment, you should identify the statistical tests used, exact values of N, definitions of center, methods of multiple test correction, and dispersion and precision measures (e.g., mean, median, SD, SEM, confidence intervals; and, for the major substantive results, a measure of effect size (e.g., Pearson's r, Cohen's d)
- Report exact p-values wherever possible alongside the summary statistics and 95% confidence intervals. These should be reported for all key questions and not only when the p-value is less than 0.05.

Please outline where this information can be found within the submission (e.g., sections or figure legends), or explain why this information doesn't apply to your submission:

Full description and justification of the statistical analyses and model fits is provided in detail in *Methods – Encoding rules and model fits* section (pp. 22-25, main text).

Statistical inference at the individual level is presented in Supplementary Figures 3 and 5.

Definition of descriptive statistics illustrating our main results is provided in the legends of Figures 4 and 5

(For large datasets, or papers with a very large number of statistical tests, you may upload a single table file with tests, Ns, etc., with reference to sections in the manuscript.)

Group allocation

- Indicate how samples were allocated into experimental groups (in the case of clinical studies, please specify allocation to treatment method); if randomization was used, please also state if restricted randomization was applied
- Indicate if masking was used during group allocation, data collection and/or data analysis

Please outline where this information can be found within the submission (e.g., sections or figure legends), or explain why this information doesn't apply to your submission:

Participants were randomly assigned to each experiment (information provided in p. 20, *Methods – participants* section)

No masking was used during group allocation, data collection and/or data analysis

Additional data files ("source data")

- We encourage you to upload relevant additional data files, such as numerical data that are represented as a graph in a figure, or as a summary table
- Where provided, these should be in the most useful format, and they can be uploaded as "Source data" files linked to a main figure or table
- Include model definition files including the full list of parameters used
- Include code used for data analysis (e.g., R, MatLab)
- Avoid stating that data files are "available upon request"

Please indicate the figures or tables for which source data files have been provided:

Hereby we confirm that raw data and essential code for data analyses will be made available on github upon acceptance of the manuscript (Information provided in p. 25, main text). We are happy to make these files available beforehand if required by the editors.
